# The effect of sexual education on the postpartum women’s sexual self-confidence and self-efficacy: a theory-based intervention

**DOI:** 10.1186/s12884-024-06255-y

**Published:** 2024-01-13

**Authors:** Mana Musavi, Mitra Rahimzadeh, Monirolsadate Hosseini Tabaghdeh, Sara Esmaelzadeh Saeieh

**Affiliations:** 1 Midwifery department, Alborz University of Medical Sciences, Karaj, Iran; 2 Social Determinants of Health Research Center, School of Public Health, Alborz University of Medical Sciences, Karaj, Iran; 3 Departments of Midwifery, Health Reproductive Research Center, Sari Branch, Islamic Azad University, Sari, Iran; 4 Social Determinants of Health Research Center, Associate Professor of Reproductive Health, Alborz University of Medical Sciences, Karaj, Iran

**Keywords:** Self-concept, Self-efficacy, Sexual self-confidence

## Abstract

**Introduction:**

Pregnancy, childbirth, and the postpartum period cause significant physical and psychological changes in mothers, leading to changes in their sexual self-concept and adverse effects on their sexual self-confidence and self-efficacy. Therefore, this study aimed to determine the effect of sexual education on postpartum women’s sexual self-efficacy and self-confidence.

**Method:**

This randomized interventional study was conducted on 115 women who had given birth at least six weeks ago and attended healthcare centers. Using convenience sampling, the researchers randomly allocated the participants into two intervention and control groups through a block size of six. The intervention group received six 90-minute online training sessions based on the sexual self-concept model over one month, while the control group received routine care. To collect data, the self-efficacy and sexual confidence questionnaires by Buzwell and Rosenthal were used before the intervention, immediately after the intervention, and one month later.

**Results:**

The study findings demonstrated no significant differences in demographic characteristics, sexual self-confidence, and sexual self-efficacy scores between the two groups before the intervention. However, the repeated measures ANOVA results revealed a substantial increase in sexual self-confidence and self-efficacy scores over time in the intervention group immediately after participating in the training sessions and one month later.

**Conclusion:**

Considering the effect of training based on the sexual self-concept model on postpartum women, the researchers recommend using this model to improve their sexual self-efficacy and self-confidence after childbirth.

**Clinical trial registration:**

This study is registered at the Iranian Registry Clinical Trial (IRCT20220530055025N1).

## Introduction

Sexual self-concept (SSC) includes all feelings and perceptions about one’s sexual relationships and self-awareness. There is a deep and significant relationship between sexual behavior and SSC; therefore, all experiences from life stages, such as pregnancy, childbirth, and postpartum, can affect SSC [1]. Chia Chun et al. revealed that SSC fundamentally moderates one’s sexual relationships and function [2]. Hence, women of reproductive age, especially postpartum, should be prioritized for sexual health education services [3].

Sexual self-confidence is an individual’s ability to experience their sexual desire and feelings comfortably and to have sexual awareness [4]. Damaged sexual self-confidence is associated with various symptoms such as health problems, anxiety, depression, homicidal and suicidal ideations and behaviors, social isolation, repressed sexual desire, and difficulty in social relationships. If the damage to sexual self-confidence is severe, it can significantly affect an individual’s efficacy [5].

Sexual self-efficacy is a factor that most sex therapists believe is influential in evaluating and determining the nature of sexual problems [6]. It is one’s perceived ability to engage in sexual activities and be desirable to their sexual partner. High self-efficacy is associated with sexual compatibility and increased sexual activity, whereas low self-efficacy negatively impacts sexual function and is associated with high-risk sexual behaviors [7].

Significant changes in appearance and weight gain can be disapproving for some women, resulting in low body image and declining sexual self-confidence [8]. Studies have shown that women in the postpartum period have lower body satisfaction than before and during pregnancy [9]. Evidence reveals that body dissatisfaction is a risk factor for decreased breastfeeding [10] and the development of eating disorders, such as anorexia and bulimia [11]. High eating disorders and body dissatisfaction are predictors of poor sexual function, low sexual self-efficacy, and more significant cognitive distraction during sexual activity [12]. Pregnant women who experience significant body changes during pregnancy have less body satisfaction after childbirth due to their inability to achieve their initial weight and body shape [13]. Dissatisfaction with body image leads to changes in sexual self-efficacy and self-confidence, affecting social and marital relationships [14].

One of the sexual health education models is the sexual self-concept (SSC), which measures cognitive, interpersonal, behavioral, physical, and influential aspects of sexual desires, such as motivation, preference, and greater extent of factors used in other models. Furthermore, this model provides a more comprehensive view of an individual’s thoughts and feelings about their sexuality. The SSC model includes six constructs: sexual self-confidence, sexual self-efficacy, anxiety, arousal, self-exploration, and commitment [15]. This model is an expanded version of Buzwell and Rosenthal’s sexual selves model presented in 1996 [16]. After the COVID-19 pandemic, general health measurement standards, including quarantine and social distancing, have changed the standards of care between therapists and clients, increasing the need for online consultation and distance education [17].

Considering the physical, psychological, and social impact of pregnancy and childbirth on mothers, this study intended to investigate the effect of online training based on the SSC model on the sexual self-confidence and self-efficacy of postpartum mothers.

## Method

This parallel randomized intervention study was conducted on 115 primiparous postpartum women, referring to healthcare centers with at least ten mothers east of Alborz Province from May to July 2022. The inclusion criteria were being Iranian, having the delivery at least 6 weeks until 12 months ago, being able to read and write in Persian, not using tobacco or drugs, being proficient in Persian, being a primipara, and having a wanted pregnancy, a term pregnancy, a neonate without anomaly, a singleton, and no pregnancy complications, such as diabetes and preeclampsia, being sexually active, having access to a cellphone, having husband, having a heterosexual orientation, and participating in online sessions. In addition, their husbands were not affected by sexual disorders. On the other hand, the exclusion criteria encompassed the mother’s absence in more than one weekly training session, hormonal problems, use of drugs affecting sexual function, medical and psychiatric illnesses, burn lesions or scars on the body that could affect sexual self-esteem, stressful experience such as the death of an acquaintance, and hospitalization within the past six months.

The sample size was calculated based on Ziaei et al. [18], employing the following formula with a 95% confidence interval and 90% power. If we expect the sexual function score to increase by at least five units in the intervention group, a sample size of 54 participants in each group is required. Considering 10% sample attrition, 60 participants were estimated for each group.

$$n=\frac{{S}_{d}^{2}{\left({Z}_{1-\frac{\alpha }{2}}+{Z}_{1-\beta }\right)}^{2}}{{\left(d\right)}^{2}}$$

Convenient sampling was employed to determine the participants, including the mothers who had given birth at least six weeks ago and were recorded in the Iranian integrated health system SIB system. First, the researchers explained the purpose of the study and obtained oral and written informed consent from the participants. Block randomization was used to reduce bias and achieve balance in allocating participants, divided into two intervention and control groups.

There were six possible probabilities for block allocation (BBAA, AABB, ABBA, BABA, BAAB, and ABAB). At the beginning of the study, the researchers selected one of the blocks and allocated six participants. Then, they assigned the A’s to the intervention group and B’s to the control group. To observe the concealment, a person other than the research team selected the type of intervention written on the sheets of paper inside the opaque envelopes.

**Fig. 1:**
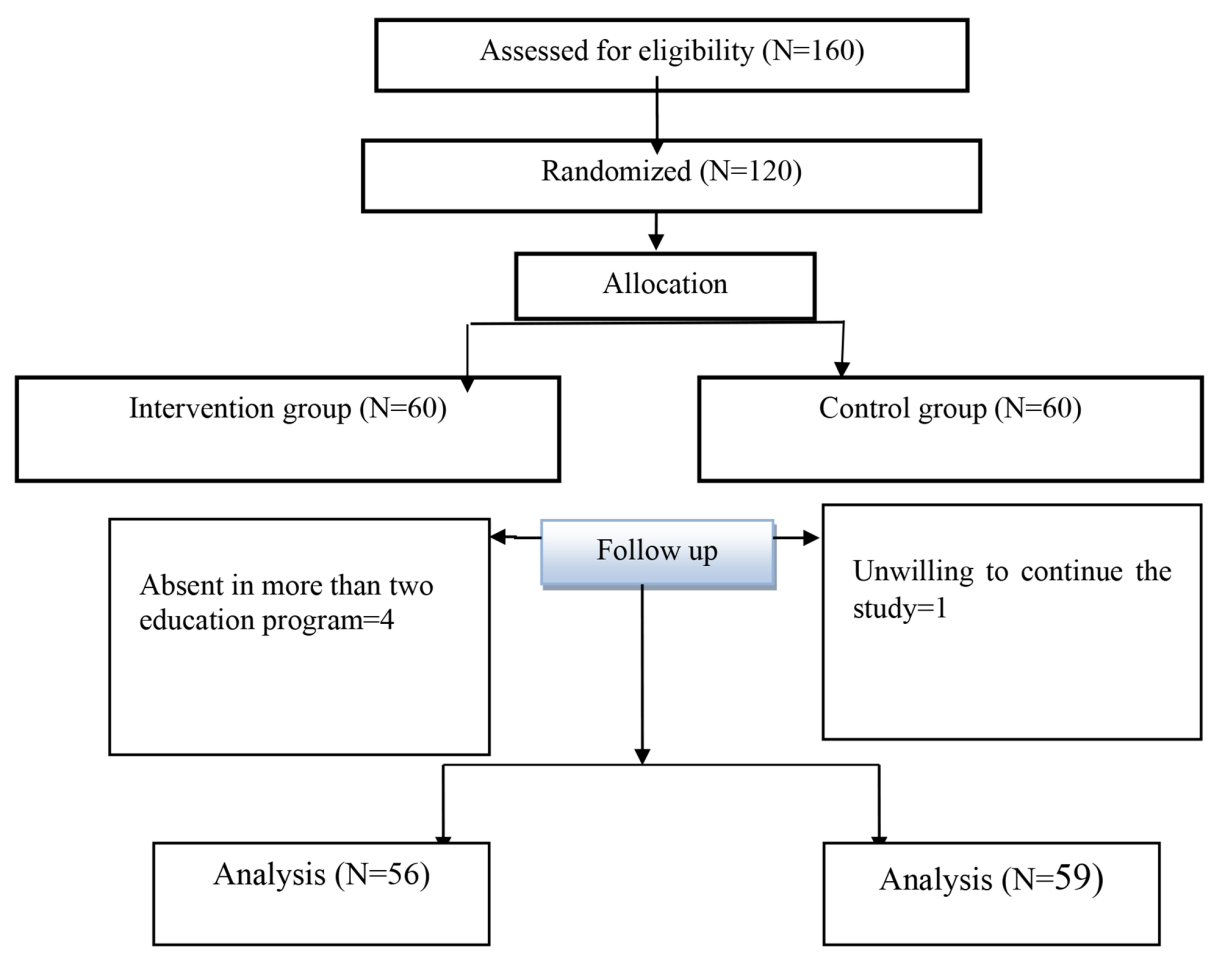
Flow Diagram of study

At the beginning of the study, participants completed the online demographic and obstetric characteristics questionnaire, sexual self-confidence, and sexual self-efficacy questionnaires. Then, the intervention group received training. Five participants were omitted from the study: four in the intervention group who did not attend all training sessions and one in the control group who did not complete the questionnaire after the intervention. Thus, data were analyzed using the pre-protocol analysis. Figure 1 shows the flow chart of the study.

The researchers conducted the training session online by presenting the slides, text files, audio recordings, and educational videos through WhatsApp and Skyroom (an Iranian platform) to communicate more with the participants. The participants could contact the researcher by cell phone if they had questions or ambiguities. The first author presented the training content online and uploaded it to WhatsApp at the end of each session. The training sessions were held in six 90-minute sessions twice weekly for one month. The intervention group received content based on the SSC model, including sexual self-confidence, sexual self-efficacy, anxiety, arousal, self-exploration, and commitment. In contrast, the control group received routine care. All participants completed the sexual self-confidence and sexual self-efficacy questionnaires immediately after the end of the training sessions and one month later to measure the educational effect over time. To observe the ethics, the education pamphlet was uploaded to WhatsApp for the control group at the end of the research. In addition, in the first session of the intervention, we asked the intervention group not to inform other pregnant mothers about the educational sessions until the end of the study to prevent sharing the content with the control group through interaction between the two groups.

The university’s midwifery and psychiatry faculty members approved the training sessions’ contents (Table 1).

**Table 1:** Contents of training sessions

**Sessions**	**Contents**
Session 1: An introduction to the course, the definition of sexual health, and SSC	The instructor explained the changes the body undergoes during the postpartum period to return to its initial state and the strategies for coping with and adapting to these changes. She also presented a definition of body image and the potential effects of pregnancy, childbirth, and the postpartum period on it, breastfeeding and its impact on physiology, appearance, and body image, and its benefits and importance. In addition, she defined SSC and explained the factors that affect its development and elements. Finally, she asked the participants about sexual relationships and the emotions they experienced, their attitudes about themselves as a sexual being, their sexual behaviors, and their perception of SSC.
Session 2: Body image and sexual self-confidence	She defined sexual self-confidence and talked about the factors such as body image affecting it, strategies to promote sexual self-confidence through relaxation, and exercises such as yoga, meditation, mental imagery, and awareness rising. In addition, she explained nutrition disorders and suggested appropriate nutritional solutions and techniques to gain fitness after pregnancy.
Session 3: Postpartum psychological issues	The instructor explained negative emotions and feelings management regarding body image, sexual self-confidence, postpartum psychological issues, including depression and its prevalence, causes of psychological problems during this period and its aggravating factors, and how to cope with and improve them. Then, she asked about psychological issues individuals have faced regarding their body image and sexual self-confidence, their mindset about sexual attractiveness, and changes in their body perception now and before pregnancy. Finally, she explained strategies to enhance their mental and psychological well-being and increase their sexual self-confidence.
Session 4: Explaining the cycle of human sexual response, anxiety, and sexual intimacy	In this session, she talked about the sexual response cycle in women and men, the factors affecting it, and disorders in the stages of the sexual cycle, excitement, plateau, orgasm, and resolution. Moreover, she explained how to understand one’s mindset regarding sexual relationships, how to initiate sexual relationships, factors affecting the sexual response cycle, one’s emotions regarding self-perception in sexual relationships, the impact of pregnancy and childbirth on it, and the role of the sexual partner in sexual relationships. The other issues discussed in this session included factors affecting sexual anxiety and creating negative feelings towards sex and sexual relationships, sexual myths and misconceptions and their role in creating unrealistic expectations in spouses and sexual anxiety, the role of sexual intimacy and communication in reducing sexual anxiety, different types of sexual, emotional, and verbal intimacy and how to use them, communication skills and techniques to increase intimacy and sexual satisfaction among couples.
Session 5: SSC and responsive sexual behavior	She defined SSC and the factors influencing it, the relevant sexual dimensions, and the techniques to improve it. In addition, she presented an explanation about how to talk with a sexual partner, express sexual expectations and desires, and control abuse in sexual relationships. She elaborated on the concept of sexual assertiveness, how to refuse unwanted sexual activity and defend one’s rights. Finally, she provided information about contraception methods, choosing the best one, and techniques to care for the baby during a sexual relationship.
Session 6: Sexual self-exploration, commitment, and summary of the previous sessions	She defined sexual self-exploration and presented strategies for creating variety and creativity in sexual relationships, which play an essential role in sexual self-efficacy. Moreover, she talked about the impact of improving sexual self-efficacy and self-confidence on sexual, marital, and overall life satisfaction.

### Instruments

This study employed the demographic and obstetric characteristics questionnaire and the sexual self-confidence and sexual self-efficacy questionnaires modified by Buzwell and Rosenthal [19]. The participants completed it before training, immediately after training, and one month later.

The questionnaires employed in this study were part of the 77-item Buzwell and Rosenthal questionnaire, which was used after confirming the content validity and confirmatory factor analysis using the Partial Lease Square software (PLS). The Sexual-Confidence Questionnaire by Buzwell and Rosenthal consisted of 24 questions, which were reduced to 19 after being checked for content analysis by ten faculty members. In addition, they removed the questions related to extramarital sex from the questionnaire because of Iranian culture. This questionnaire measures individuals’ sexual self-confidence based on a 5-point Likert scale ranging from strongly disagree to strongly agree. The questionnaire has four components, including sexual behavior with five items (α = 0.82), sexual attractiveness with five items (α = 0.79), sexual guidance with four items (α = 0.82), and physical perception with five items (α = 0.82); all components have sufficient reliability according to Cronbach’s alpha coefficient. PLS software was employed to determine its construct validity; all the remaining items had a factor loading of > 0.7 and remained in the questionnaire.

### Sexual self-efficacy questionnaire

This part of the Buzwell and Rosenthal [19] questionnaire includes three subscales: six questions to measure perceived responsibility about sexual activity (α = 0.86), the sexual assertive subscale with six items to evaluate the ability to achieve sexual satisfaction (α = 0.68), and precaution subscale with eight items regarding the ability to purchase and use condoms (α = 0.68). The questionnaire consists of 20 questions on a 5-point Likert scale ranging from very uncertain to absolutely certain. Ten faculty members analyzed the questionnaire and reduced the number of questions to 14; they removed the questions about extramarital sex. PLS software was used to determine the construct validity; none of the remaining questions had a factor load of < 0.7 and remained in the questionnaire.

### Statistical analysis

After data collection, the data were fed into SPSS-16, and then the normality of the data was determined by skewness and kurtosis. Skewness and kurtosis were lower than 1.8, showing a normal variable distribution. The missing data were indicated and replaced with the median. Socio-demographic and obstetrics characteristics were examined using independent t-tests, fisher, and chi-square tests. The repeated measure ANOVA test showed a trend of sexual self-efficacy and self-confidence variables and compared the two groups.

## Results

The study results show that the mean age of the participants is 31.4 ± 6.09 in the intervention group and 29.06 ± 6.48 in the control group. The percentages of participants with a diploma or less education level were 75.6% in the intervention group and 74.5% in the control group. In the intervention group, 80.3%, and in the control group, 74.5% of mothers were housewives. Table 2 summarizes the demographic characteristics of mothers participating in the study. There is no statistically significant difference between demographic characteristics in the control and intervention groups.

**Table 2:** Demographic characteristics of the study participants

variable	Intervention group F( Percent)	Control group F( Percent)	*p*-value	Statistical test
**Woman age)Mean± SD)**	31.4 ± 6.09	29.06 ± 6.48	0.055	T-Test
**Education**			0.25	Fisher
High school	23(41.7)	16(27.1)		
High school diploma	19(33.9)	28(47.4)		
University	14(24.9)	15(25.3)		
**Job**			0.54	Chi-square
Housewife	45(80.3)	44(74.5)		
Part-time Employee	2(3.6)	4(6.8)		
Full-time Employee	9(16.1)	11(18.7)		
**Socio-economic status**			0.62	Fisher
Weak	17(30.3)	14(23.7)		
Intermediate	36(64.2)	43(72.8)		
Good	3(5.3)	2(3.3)		
**Type of delivery**				
Cesarean	26(46.4)	29(49.1)	0.71	Chi-square
Vaginal delivery	33(53.6)	27(40.9)		
Assisted vaginal delivery (e.g., forceps, vacuum)	0	0		
**Baby Sex**			0.45	Chi-square
Male	30(53.3)	27(45.7)		
Female	26(46.7)	32(54.3)		
**Number of children**			0.61	Chi-square
1	16(28.5)	22(37.2)		
2	21(37.5)	20(33.8)		
3	15(26.7)	11(18.6)		
4	4(7.1)	6(10.1)		

The repeated measure ANOVA was run to examine the effect of SSC model training on the total score of sexual self-efficacy before, after, and one month after the intervention. Since the significance level of Mauchly’s test is less than 0.05, the result of the repeated measure ANOVA with the Greenhouse-Geisser assumption indicates the change of the sexual self-efficacy score and its dimensions over time in the group receiving training. Moreover, the two groups have a significant difference regarding the sexual self-efficacy score and its dimensions (Table 3).

**Table 3:** The repeated measure ANOVA for Sexual self-efficacy score before, after, and one month after the intervention

	Group	Mean ± SD Before	Mean ± SD After	Mean ± SD One month after	Repeated	measure		
Factor							Eta	Muchly
					Within group	Between group		
sexual assertive	Intervention	9.8 ± 2.2	18.01 ± 1.3	17.08 ± 1.6	F = 423.3 *P* > 0.001	F = 246.1 *P* > 0.001	0.64	0.87 *P* > 0.001
	control	10.3 ± 3.1	10.6 ± 3.03	11.9 ± 2.3				
Sexual Satisfaction	Intervention	15.01 ± 3.1	22.1 ± 2.1	23.3 ± 1.3	F = 339.2 *P* > 0.001	F = 135.6 *P* > 0.001	0.54	0.83 *P* > 0.001
	control	14.9 ± 3.9	15.7 ± 3.3	17.3 ± 2.8				
Precaution	Intervention	15.3 ± 4.2	26.8 ± 2.07	25.5 ± 2.8	F = 277.1 *P* > 0.001	F = 177.3 *P* > 0.001	0.72	0.82 *P* > 0.001
	control	14.3 ± 5.05	14.6 ± 4.7	16.4 ± 3.5				
Total score	Intervention	53.3 ± 6.06	8.07 ± 7.1	83.6 ± 5.9	F = 266.6 *P* > 0.001	F = 244.3 *P* > 0.001	0.07	0.74 *P* > 0.001
	control	54.6 ± 8.5	53.9 ± 8.9	56.3 ± 7.5				

Another repeated measure ANOVA was calculated to investigate the effect of SSC model-based training on the total sexual self-confidence scores before, after, and one month after the intervention. Since the significance level of Mauchly’s test is less than 0.05, the results of the repeated measure ANOVA with the Greenhouse-GEISSER assumption illustrate the change in the sexual self-confidence score and its dimensions over time in the group receiving training. Moreover, there is a significant difference between the two study groups regarding the total sexual self-confidence score and its dimensions (Table 4).

**Table 4:** The repeated measure ANOVA for the sexual self-confidence score before, after, and one month after the intervention

	Group	Mean ± SD Before	Mean ± SD After	Mean ± SD One month after	Repeated	measure		
Factor							Eta	Muchly
					Within group	Between group		
Sexual guidance	Intervention	10.5 ± 2.07	17.4 ± 2.01	16.5 ± 2.2	F = 101.3 *P* > 0.001	F = 19.4 *P* > 0.001	0.43	0. 7 *P* > 0.001
	control	11.7 ± 3.07	11.6 ± 2.6	13.1 ± 2.9				
Physical perception	Intervention	14.4 ± 2.2	21.7 ± 2.1	22.6 ± 1.8	F = 333.4 *P* > 0.001	F = 214.6 *P* > 0.001	0.65	0.83 *P* > 0.001
	control	16.3 ± 2.9	16.3 ± 2.7	17.7 ± 3.6				
Attractiveness	Intervention	14.5 ± 3.4	20.7 ± 2.4	21.4 ± 2.1	F = 108.9 *P* > 0.001	F = 82.09 *P* > 0.001	0.42	0.84 *P* > 0.001
	control	15.1 ± 3.3	14.9 ± 3.5	16.3 ± 2.9				
Behavior	Intervention	14.7 ± 3.8	14.8 ± 3.2	14.7 ± 3.8	F = 242.8 *P* > 0.001	F = 135.8 *P* > 0.001	0.54	0.88 *P* > 0.001
	control	14.2 ± 2.5	22.5 ± 1.8	21.7 ± 2.1				
Total score	Intervention	38.2 ± 6.6	60.3 ± 51	63.8 ± 3.4	F = 198.6 *P* > 0.001	F = 173.6 *P* > 0.001	0. 6	0.61 *P* > 0.001
	control	37.6 ± 9.5	94 ± 7.06	40.7 ± 7.4				

## Discussion

The results of the present study demonstrated that the mean self-efficacy score in the intervention group was higher than the control group after the intervention and one month later. Therefore, training based on the SSC model positively affected the sexual self-efficacy of women who have given birth to a child.

Several studies have shown that different psychological approaches improve women’s sexual function by promoting their sexual self-efficacy [20–22]. In this regard, Ziaei et al. revealed that SSC-based counseling increased women’s sexual self-efficacy and improved their sexual function [18]. Similar to Atrinan et al., they demonstrated that with training, the sexual self-efficacy score in the dimensions of sexual assertiveness, sexual satisfaction, and condom use had increased significantly, leading to improved sexual satisfaction and sexual function in women [23]. Many studies examined the role of using the SSC model to reduce sexual risk-taking and found a significant relationship between improving SSC and reducing sexual risk-taking. Reducing sexual risk-taking includes dimensions such as condom use and sexual resistance; the study results showed improvement in the scores of these dimensions through training based on the SSC model. Improving sexual self-efficacy in these dimensions makes women take responsibility for their sexual relationships and refuse them freely when they do not want to keep a sexual relationship or a particular type of sexual relationship [15].

In this study, the result of the repeated measurement ANOVA illustrated a change in the sexual self-confidence score over time in the intervention group and between the two groups. In one month of counseling in the intervention group, the sexual self-confidence score in the control group, receiving the routine care, had decreased significantly compared to before the study. It could be due to the factors that reduce sexual self-confidence after childbirth, such as body dissatisfaction, changes in body perception, sexual attractiveness, and sexual anxiety. Although the sexual self-confidence score approached the initial score over time in the control group after a month, the results illustrated that it was insignificant. On the other hand, the sexual self-confidence score and its dimensions significantly improved immediately after counseling and one month later in the intervention group. Moreover, Hensel et al. showed that the proper evolution of SSC resulted in less anxiety, greater comfort with sexuality, and better sexual behavior [24].

Training based on psychological approaches, including the SSC model, with different sexual dimensions, significantly impacts people. One of the essential dimensions of sexual self-confidence is body perception; greater body perception has many benefits, including increasing body satisfaction [16]. People with a more positive body perception felt more sexually attractive and showed better sexual behavior [25]. On the other hand, Talebi et al. [26] and Sabaghian et al. [27] showed that psychological training did not have an influential role in body satisfaction. They believed that the difference in the results of the studies was because of the type of training approach, content, and intervention implementation quality. According to the results of the present study, the SSC model positively influenced different sexual dimensions. Due to the interaction between these dimensions, the score in each dimension improved.

High sexual self-confidence and proper sexual function play a significant role in increasing marital satisfaction and consequently improve the quality of life. People with high sexual self-confidence can establish happy and successful relationships with their sexual partners. They can control their feelings and sexual relationships and reduce their fears; therefore, they have more desire to have sex and experience higher sexual satisfaction [28]. In contrast, low sexual self-confidence can disturb life satisfaction and self-view. If the damage to self-confidence is severe, it can cause severe sexual dysfunction [29]. Therefore, considering the physical and psychological changes in women’s bodies following childbirth, improving sexual self-confidence can increase marital and general life satisfaction.

Screening and diagnosing the problems of sexual self-concept, self-confidence, and sexual self-efficacy of people play an important role in improving interpersonal relationships and sexual communication. Pregnancy and the postpartum period affect the sexual self-concept, self-confidence, and sexual self-efficacy of women and change their sexual health and quality of life. Therefore, it is very important to identify and understand the causes of the disorder, as well as to understand the educational needs and empowerment of such women.

Therefore, according to the role of midwives in postpartum care, it is suggested that they evaluate the sexual self-concept, self-confidence, and sexual self-efficacy of mothers, and if necessary, plan the educational program based on the model of sexual self-concept, culture, and race of the relatives to provide them with the necessary knowledge, attitudes, and related skills to improve the mother’s self-confidence, sexual self-efficacy, and quality of life.

One of the limitations of the current research was the online training course, so people with better economic and social status took part in the study. On the other hand, being an online course could be the study’s strength. According to the participants’ point of view, they could talk about their experiences and problems freely without embarrassment, and issues such as sexual training could be expressed more easily. This study was conducted on middle-income Iranian women referred to the public health center of one province of Iran.

In this study, the effect of sexual orientation on self-concept, self-confidence, and sexual self-efficacy was not investigated because they are taboo in Iranian culture, and many people do not like to talk about them openly. In future studies, the effect of sexual orientation on sexual concepts, sexual confidence, and sexual efficacy should be investigated. The findings are less likely to generalize to women of higher socioeconomic status or other ethnicities.

## Conclusion

Training based on the SSC model positively affected sexual self-efficacy and self-confidence in postpartum women, directly associated with improved sexual function and higher marital satisfaction. Therefore, the researchers recommend that healthcare-providing centers, including public healthcare centers, hospitals, and private healthcare centers, hold training courses based on this model to improve the sexual function of the women following childbirth and influence their sexual self-confidence and self-efficacy.

## Data Availability

The datasets generated and analyzed during the current study are not publicly available due to the confidentiality of information. Still, they can be available through the corresponding author upon reasonable request.
